# V-Doped CoP Nanosheet Arrays as Highly Efficient Electrocatalysts for Hydrogen Evolution Reaction in Both Acidic and Alkaline Solutions

**DOI:** 10.3389/fchem.2020.608133

**Published:** 2020-10-23

**Authors:** Wei Hua, Huanhuan Sun, Lingbo Ren, Ding Nan

**Affiliations:** ^1^State Key Laboratory of Solidification Processing, Center for Nano Energy Materials, School of Materials Science and Engineering, Northwestern Polytechnical University, Shaanxi Joint Lab of Graphene (Northwestern Polytechnical University), Xi'an, China; ^2^School of Materials Science and Engineering, Inner Mongolia University of Technology, Hohhot, China

**Keywords:** hydrogen evolution reaction, V-doped CoP, acid and alkaline solutions, nanosheet arrays, water splitting

## Abstract

It is of significant necessity to explore inexpensive and high-active electrocatalysts toward hydrogen evolution reaction (HER) in both acidic and basic media. In this work, V-doped CoP nanosheet arrays supported on the carbon cloth (V-CoP/CC) are fabricated though a facile water-bath/phosphorization method. The nanoarray structure on the three-dimensional self-supporting electrode can provide a large electrochemical active surface area with more exposed active sites to accelerate the reaction kinetics. Furthermore, V doping is able to tune the electronic properties and thus enhance the intrinsic catalytic activity of CoP. Consequently, the V-CoP/CC electrode exhibits excellent electrocatalytic activities toward HER in both 0.5 M H_2_SO_4_ and 1 M KOH solutions with small overpotentials of 88 and 98 mV at a current density of 10 mA cm^−2^, respectively. The present work will offer a feasible way to tailor the catalytic activity by hetero-atoms doping toward HER.

## Introduction

Hydrogen is an effective energy carrier to solve the contemporary energy crisis and environmental pollution (Luo et al., 2014; Seh et al., 2017). Nowadays, hydrogen is still produced mainly from fossil fuels, which suffers from emission of large quantities of carbon dioxide (Zou and Zhang, 2015). Electrolysis of water based on renewable resources is considered as an practical method for producing hydrogen sustainability (Li and Zheng, 2017; Hua et al., 2020). By far, the state-of-the-art electrocatalysts for hydrogen evolution reaction (HER) are still precious Pt-based materials, the scale-up application of which, however, is restricted to their scarcity and high cost. Therefore, the development of low-cost and high-activity electrocatalysts for HER has attracted a wide spread attention.

The earth-abundant transitional metal-based materials, such as alloys (Zhang et al., 2017), oxides (Dong et al., 2019), sulfides (Chen et al., 2019), selenides (Liu et al., 2017), carbides (Lu et al., 2019), nitrides (Yao et al., 2019) and phosphides (Wang J. et al., 2018), have been proved as promising catalysts for HER. Of particular note, more efforts have been made in the design and preparation of transitional metal phosphides (TMPs) as HER catalysts owing to the excellent electrocatalytic performance and long stability in both acidic and alkaline media (Shi and Zhang, 2016; Wang Y. et al., 2017). However, the electrochemical activity of monophase TMPs needs to be further improved compared with precious metal-based materials. HER occurs at the surfaces of electrocatalysts, hence, increasing the number of active sites is of great benefit in improving the catalytic performance. Notably, the array architecture combined with a bind-free three-dimensional (3D) self-supporting electrodes displays much better HER properties than the planar electrodes due to the more accessible active sites (Wang Y. et al., 2018). Moreover, hetero-atom doping has been an effective method to improve the performance of electrocatalysts. For example, Pan et al. (2016) investigated the doping effect of Fe, Ni, and Cu elements on the catalytic properties of the Co_2_P catalysts. Electrochemical results show that the electrocatalytic performance for HER follows the trend of Fe-Co_2_P > Ni-Co_2_P > Cu-Co_2_P in the acidic solution, which is related to the difference of morphology, and electronic structure after doping. Recently, Wang's group reported a Bi/CoP sample with a flower-like structure (Guo et al., 2020). The theoretical calculation revealed that Bi doping could tune the hydrogen binding energy of CoP, enabling the Bi/CoP electrode to display high electrocatalytic properties for HER in both alkaline and acidic media. In addition, doping with various metal elements, such as Er (Zhang et al., 2019), Al (Du et al., 2018), Mo (Guan et al., 2018), etc., has been reported with enhanced electrocatalytic activities. To our knowledge, the V doping in TMPs has barely been reported. Therefore, it is highly imperative to design and investigate the effect of V doping on the performance for HER.

Herein, the influence of V doping on the electrocatalytic activity of CoP toward HER is investigated and identified in both acidic and basic media. The V-doped CoP nanosheet arrays grown on carbon cloth (CoP/CC) have been prepared though a facile water bath/phosphorization approach. Owing to the 3D conductive CC substrate, nanosheet array structure, and the optimized electronic structure by V doping, CoP/CC exhibits much enhanced electrocatalytic HER activity with low overpotentials of 88 and 98 mV at 10 mA cm^−2^ in 0.5 M H_2_SO_4_ and 1 M KOH, respectively. The excellent performance indicates that V doping is an efficient strategy to improve the performance of HER electrocatalysts in both acidic and alkaline solutions.

## Experimental Section

### Materials Synthesis

Prior to synthesis, a piece of carbon cloth was treated in 6 M nitric acid at 85°C overnight. For the preparation of cobalt-based metal-organic frameworks on CC (CoMOF/CC), 2 mmol cobalt (II) nitrate hexahydrate [Co(NO_3_)_2_·6H_2_O] and 16 mmol 2-methylimidazole (2-mim) were dispersed in 40 ml deionized water (18.2 MΩ), respectively. Then the homogeneous aqueous solution of 2-mim was added into the Co(NO_3_)_2_ solution and a piece of treated CC was put into the above mixture for 2 h at room temperature. For the synthesis of V-Co layered double hydroxide (LDH), the as-prepared CoMOF was immersed into 50 ml water/ethanol solution (4:1 in volume) containing 100 mg sodium orthovanadate (Na_3_VO_4_), then it reacted at 50°C for 20 min. Finally, the V-Co LDH grown on the CC and 0.2 g NaH_2_PO_2_ placed at the middle of tube furnace, with NaH_2_PO_2_ at the upstream side, which were then heated to 350°C with a ramping rate of 2°C min^−1^ under Ar flow and kept for 2 h. The mass loading of the as-synthesized V-CoP is about 2.4 mg cm^−2^. For the preparation of hollow CoP, CoMOF was annealed for 1 h at 300°C with a heating rate of 1°C min^−1^ under air conditions and then the phosphidation procedure was similar to that of V-CoP. The mass loading of CoP is about 2 mg cm^−2^.

### Materials Characterization

Field-emission scanning electron microscopy (SEM, FEI NanoSEM 450) and transmission electron microscopy (TEM, FEI Talos F200X) were used to study the morphology and structure of the as-synthesized samples. The phased structure was identified by X-ray diffraction (XRD, Shimadzu XRD-7000).

### Electrochemical Evaluation

Electrochemical performance of the electrodes was tested on an electrochemical workstation (CHI 660E). The as-received samples and carbon rod were served as the working electrode and counter electrode, respectively. The reference electrodes were Hg/HgO electrode and saturated calomel electrode (SCE) in 1.0 M KOH (pH = 13.8) and 0.5 M H_2_SO_4_ (pH = 0.6), respectively. Polarization curves were tested by linear sweep voltammetry (LSV) at 2 mV s^−1^, and the potential values were calibrated to reversible hydrogen electrodes (RHE) with IR-correction, E_RHE_= E_Hg/HgO(SCE)_ + 0.097 + 0.059 pH. The IR-correction was conducted by E_Corrected_ = E_Raw_ - IRs, here, Rs corresponds to the series resistance, which can be obtained from the electrochemical impedance spectrum (EIS) measurements. The durability of the sample was studied by chronopotentiometry method at a constant current density of 10 mA cm^−2^ and cyclic voltammetry (CV) at a scan rate of 50 mV s^−1^ for 2,000 cycles. EIS was measured at −0.1 V vs. RHE in the frequency range between 0.1 Hz and 100 KHz with an AC amplitude of 5 mV. CV measurement was tested in the potential range from 0.15 to 0.25 V vs. RHE at various scan rates to estimate the electrochemical active surface area (ECSA).

## Results and Discussions

**Figure 1:**
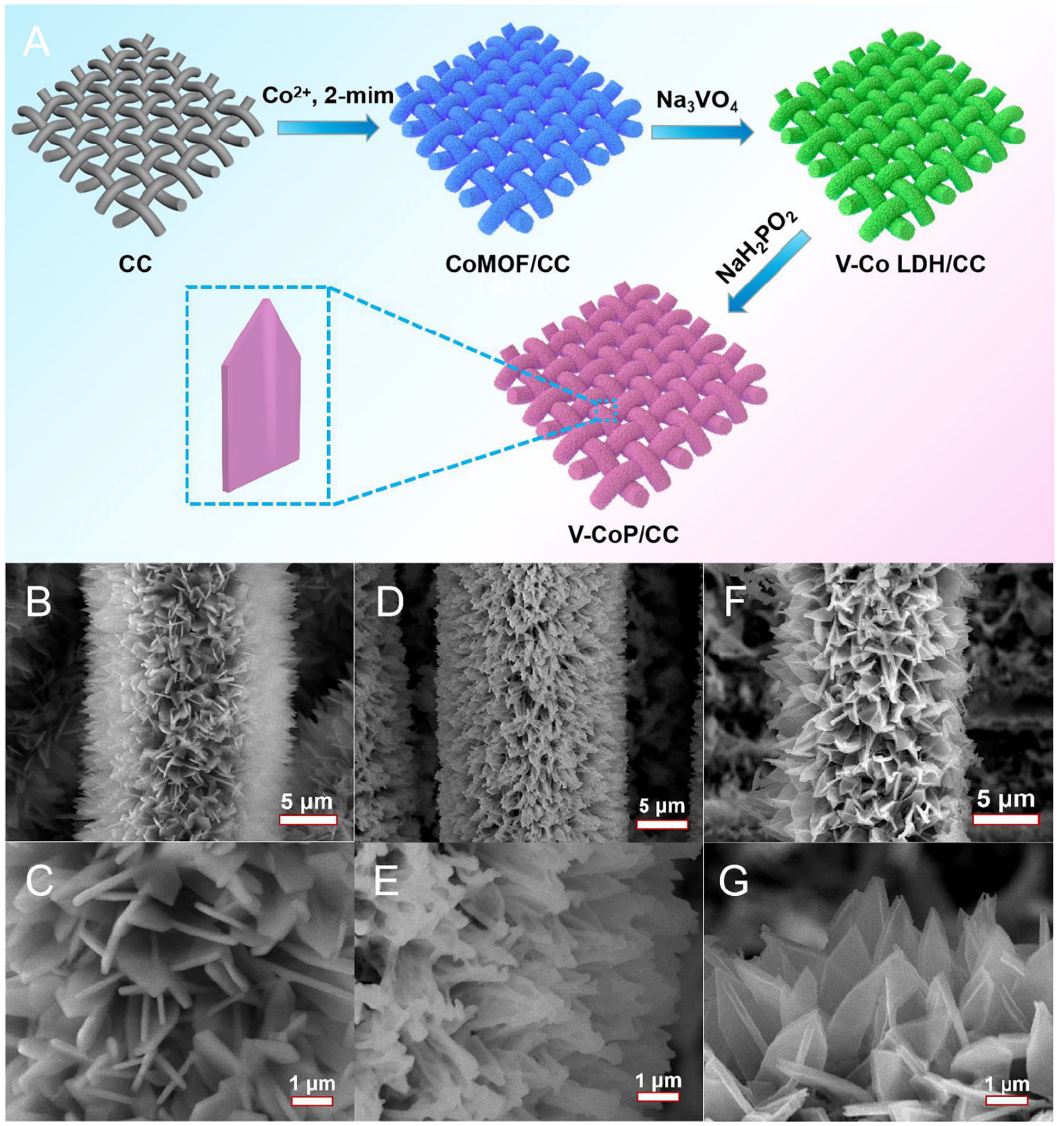
**(A)** Schematic illustration of the fabrication process of V-CoP nanosheet arrays on the CC. SEM images of CoMOF **(B,C)**, V-Co LDH **(D,E)**, V-CoP **(F,G)** on the CC.

The preparation process of the V-CoP/CC electrode is schematically illustrated in Figure 1A. Firstly, CoMOF nanosheet arrays are uniformly grown on the surface of the carbon cloth via a simple aqueous solution reaction between Co^2+^ and 2-mim at room temperature (Fang et al., 2016). The SEM images (Figures 1B,C) show the uniform CoMOF nanosheet arrays supported on the CC. Secondly, the as-synthesized CoMOF precursor was immersed into a Na_3_VO_4_ solution. Owing to the hydrolysis of the solution, the hydrogen ions generated enhanced the etch of CoMOF and release Co ions, at the same time, the local pH increased, making V and Co precipitate on its surface to form V-Co LDH (Figures 1D,E). Finally, the V-CoP/CC was obtained through a typical phosphidation process. Remarkably, as shown in Figures 1F,G, the as-prepared V-CoP well-retain the nanoarray architecture on the CC. Similarly, CoP maintains the original morphology after phosphidation (Supplementary Figures 1A,B).

**Figure 2:**
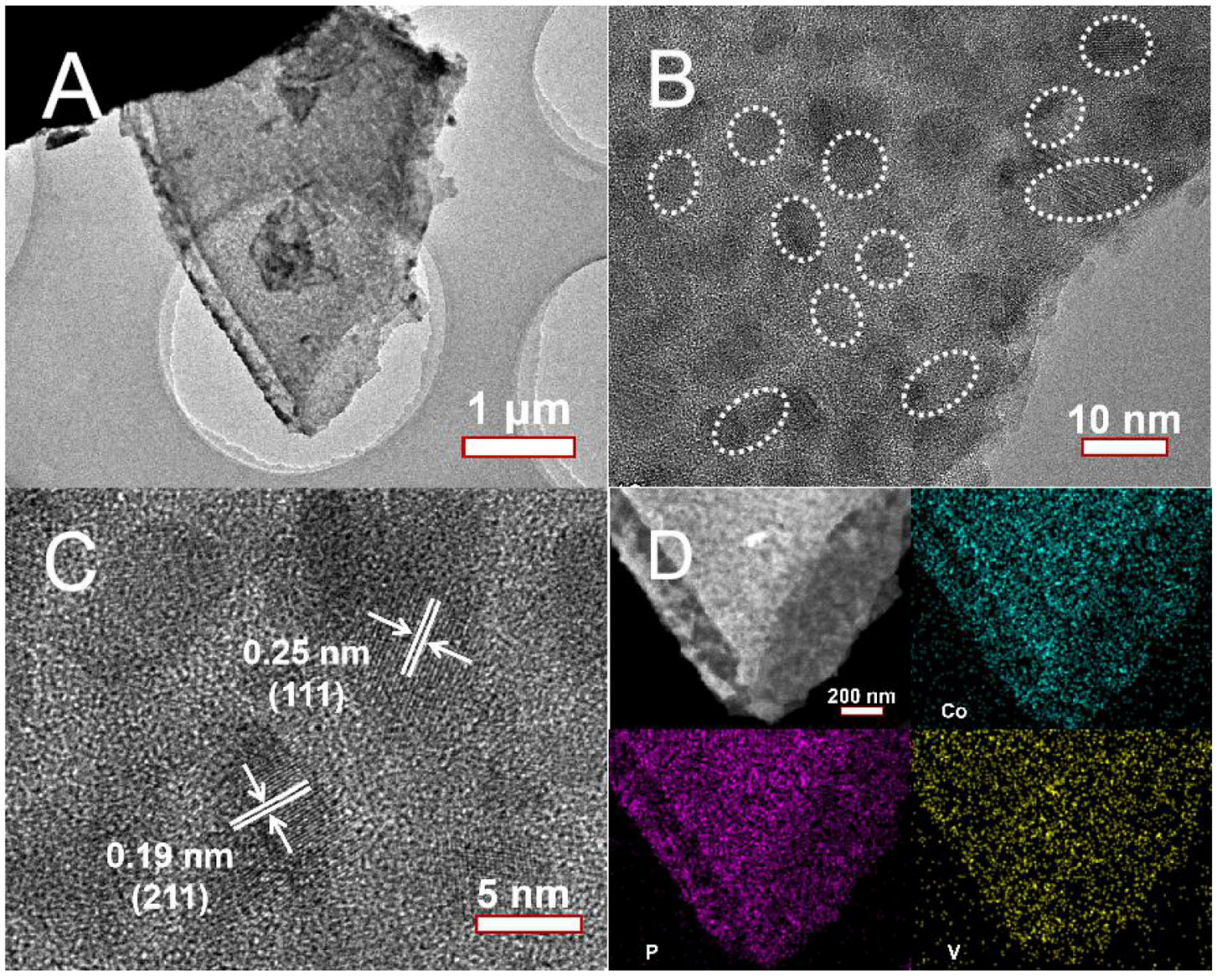
**(A,B)** TEM and **(C)** HRTEM images of V-CoP/CC, **(D)** HAADF-STEM image of V-CoP/CC and EDS elemental mappings of Co, P, and V elements.

To further investigate the microstructure, the V-CoP nanosheets are characterized by TEM imaging. As displayed in Figure 2A, the V-CoP exhibits a thin nanosheet structure. Figure 2B reveals that the nanosheet is consisted of numerous ultrafine crystalline nanoparticles and amorphous components. The HRTEM image (Figure 2C) shows distinct crystal spacings of 0.25 nm and 0.19 nm on the nanoparticles, which are indexed to the (111) and (211) facets of CoP, respectively (Wang et al., 2019). Moreover, the specific feature of V-CoP nanosheet is further examined by HAADF-STEM and energy dispersive spectrometer (EDS) elemental mapping (Figure 2D). The EDS mapping result demonstrates the homogeneous distribution of Co, P and V elements, indicating that V element is uniformly doped in CoP and the atomic ratio of V:Co is determined to be about 0.23:1. The phase structures of the as-synthesized V-CoP/CC and CoP/CC are analyzed by XRD. As displayed in Supplementary Figure 2, apart from the two diffraction peaks corresponding to the CC substrate (Guan et al., 2017), there are no other distinct diffraction peaks. The phenomenon is consistent with the result of TEM that the sample is made up of ultrathin nanoparticles and amorphous components.

**Figure 3:**
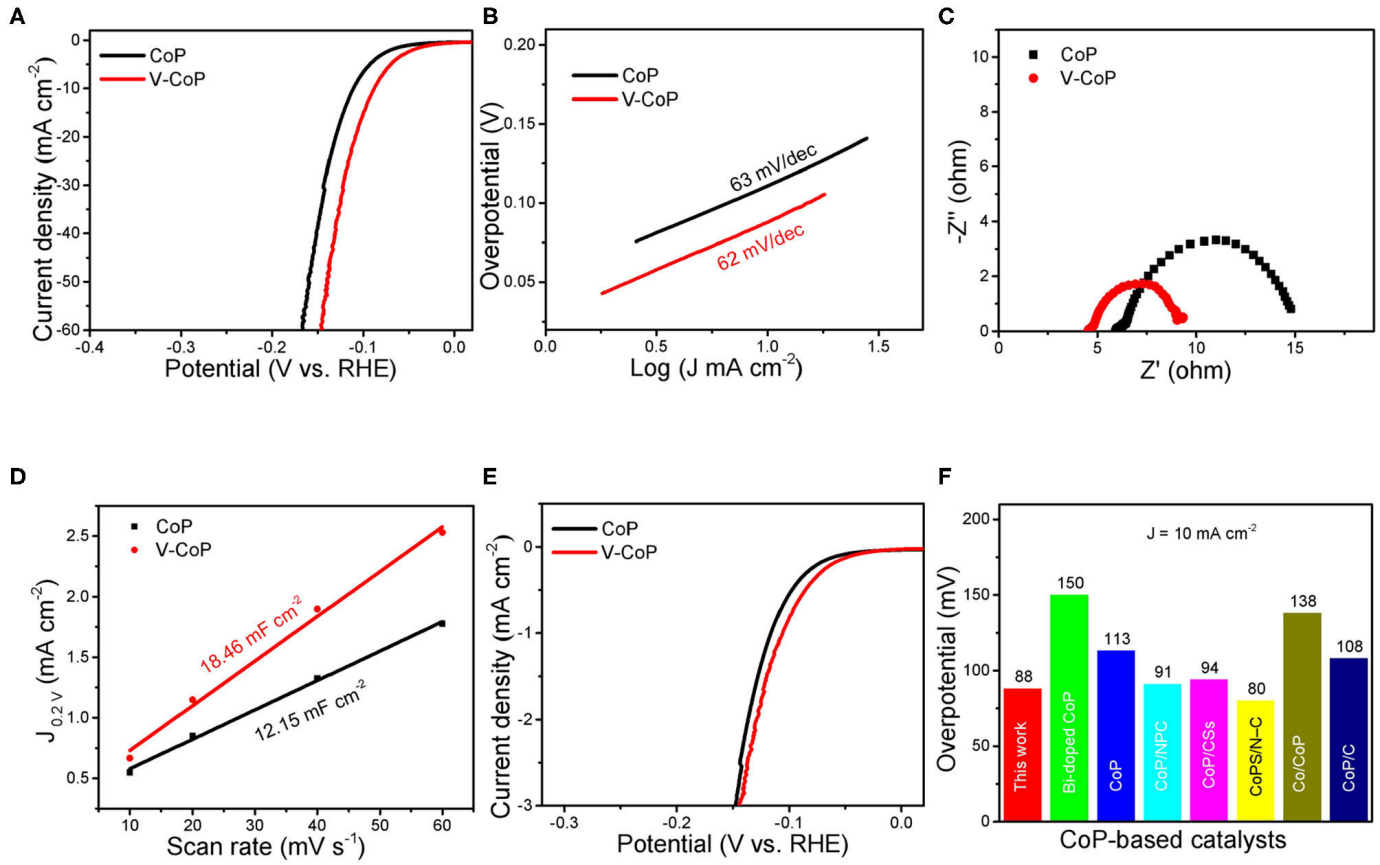
HER performance of V-CoP/CC and CoP/CC in 0.5 M H_2_SO_4_. **(A)** LSV curves, **(B)** Tafel plots, **(C)** Nyquist plots. **(D)** CV curves and estimated C_dl_ of V-CoP/CC and CoP/CC. **(E)** Polarization curves normalized to the ECSA. **(F)** A comparison of HER performance of CoP-based catalysts in 0.5 M H_2_SO_4_. NPC, N,P-doped carbon; CSs, carbon spheres; and N-C, N-doped carbon.

The results of the above characterization indicate that V-doped CoP nanosheet arrays on the CC are successfully prepared. The electrocatalytic HER performance of the sample in the acidic solution is first investigated. As displayed in Figure 3A, the CoP/CC requires an overpotential of 111 mV to afford the current density of 10 mA cm^−2^, whereas V-CoP/CC only needs 88 mV to produce the same current density. The Tafel slopes are further calculated to study the HER mechanism on the electrocatalysts (Figure 3B). The Tafel slopes of CoP/CC and V-CoP/CC are 62 and 63 mV/dec, respectively. The similar values indicate that the hydrogen evolution on the two samples follows the same Volmer-Heyrovsky mechanism. To study the kinetics of the catalysts, charge transfer resistances (R_ct_) have been characterized by EIS at −0.1 V vs. RHE. As shown in Figure 3C, the V-CoP/CC manifests a smaller value of R_ct_ (4.6 Ω) than that of CoP/CC (9 Ω), indicating a faster reaction kinetics (Sun et al., 2020). To further understand the reason for the higher elecreocatalytic activity of V-CoP/CC, the electrochemical double layer capacitances (C_dl_) is measured by CVs with various scanning rates in the potential range between 0.15 and 0.25 V vs. RHE (Supplementary Figures 3A,B). As observed in Figure 3D, the V-CoP/CC electrode shows a bigger C_dl_ (18.46 mF cm^−2^) than CoP/CC (12.15 mF cm^−2^), suggesting that V-CoP/CC has higher ECSA and is able to expose more active sites during the reaction process. To investigate the intrinsic activity of V-CoP/CC, the polarization curves are further normalized to the ECSA (Figure 3E). Notably, the V-CoP/CC displays much higher current density than CoP/CC, indicating that V doping can improve the intrinsic catalytic activity of CoP (Liang et al., 2016). The aforementioned results show that V doping can not only increase the number active sites, but also optimize the electronic structure of CoP and improve the intrinsic catalytic activity, thus exhibiting the enhanced catalytic performance toward HER in acidic media. It is worth to note that the HER performance of the present V-CoP/CC electrode outperforms those of most reported CoP-based catalysts in acidic solution, as shown in Figure 3F (Luo et al., 2017; Wang H. et al., 2017; Li H. et al., 2018; Li, Y et al., 2018; Gao et al., 2019; Huang et al., 2019).

**Figure 4:**
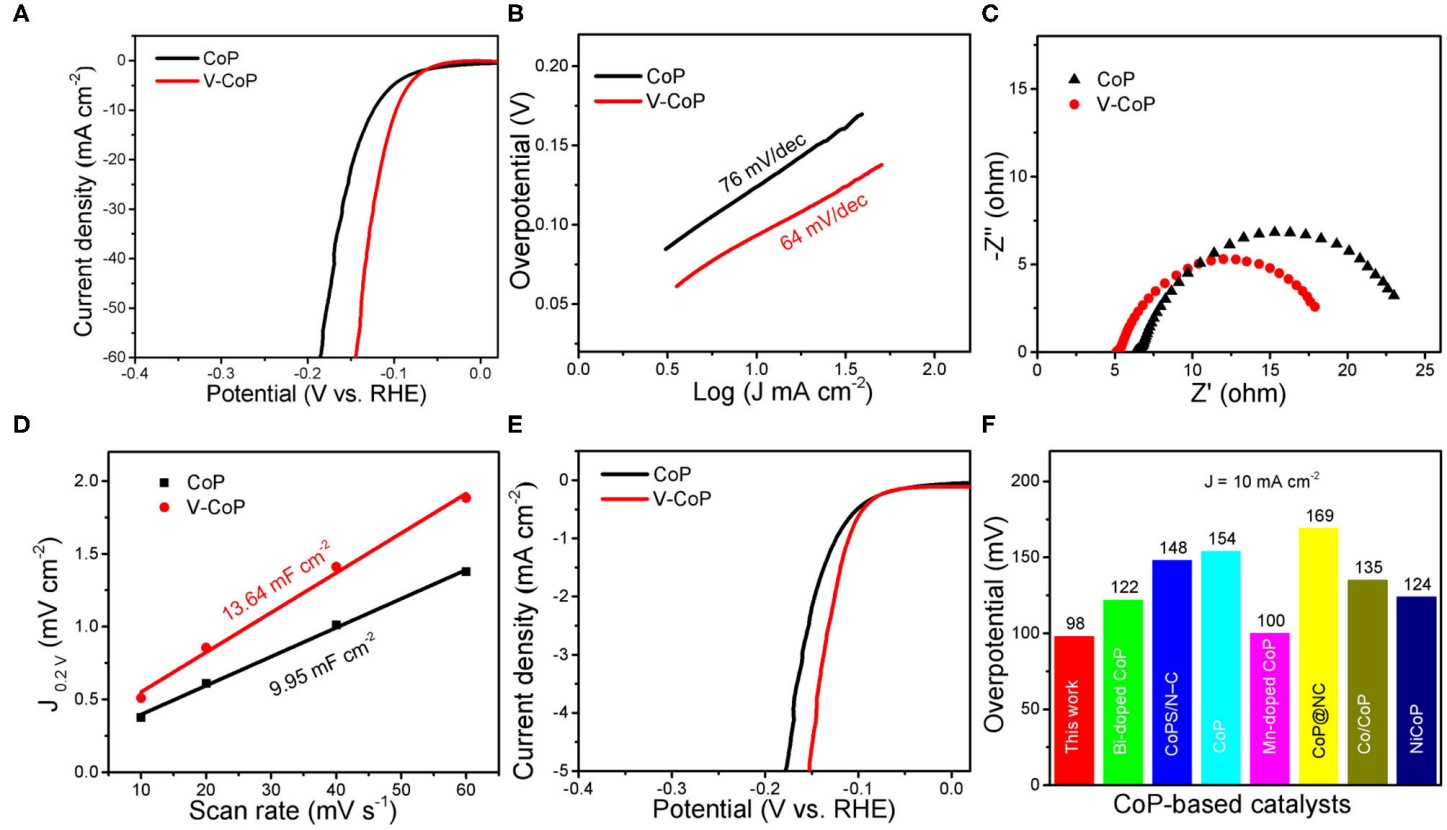
HER performance of V-CoP/CC and CoP/CC in 1 M KOH. **(A)** LSV curves. **(B)** Tafel plots. **(C)** Nyquist plots. **(D)** The estimated C_dl_ of V-CoP/CC and CoP/CC. **(E)** Polarization curves normalized to the ECSA. **(F)** A comparison of HER activities of CoP-based catalysts in 1 M KOH. NC, nitrogen-doped porous carbon.

The V-CoP/CC electrode has shown enhanced electrocatalytic performance for HER in acidic media. Meanwhile, water-alkali electrolyzer needs active and low-cost electrocatalysts that can work well in basic condition. As shown in Figure 4A, the V-CoP/CC also displays good catalytic performance toward HER in 1M KOH solution, reaching the current density of 10 mA cm^−2^ with a small overpotential of 98 mV. By contrast, the CoP/CC electrode requires a higher overpotential of 124 mV to obtain the current density of 10 mA cm^−2^. Tafel slopes are also employed to investigate the reaction kinetics in basic media. The Tafel slopes of V-CoP/CC and CoP/CC electrodes are 64 and 76 mV/dec, respectively (Figure 4B). The value of V-CoP/CC is similar with that of the electrode in acidic media, while CoP/CC exhibits higher Tafel slope in 1 M KOH, suggesting that V-doping can improve the water dissociation process and accelerate the reaction kinetics in alkaline condition. In line with this result, as displayed in Figure 4C, the V-CoP/CC shows a smaller R_ct_ (13.9 Ω) than CoP/CC (18.3 Ω). The CVs (Supplementary Figures 4A,B) in 0.15–0.25 V vs. RHE are also tested to calculate the C_dl_ (Figure 4D). The C_dl_ value of V-CoP/CC is 13.64 mF cm^−2^, which is higher than that of CoP/CC (9.96 mF cm^−2^). The current density is also normalized to the corresponding ECSA, the property of V-CoP/CC is still superior to CoP/CC (Figure 4E). It is worth to note that, when normalized to ECSA, the performance difference between CoP and V-CoP is larger in alkaline than in acidic electrolyte. HER in basic media is not only dominated by the binding energy of hydrogen, but also depends on the water dissociation step. The larger difference indicate that V doping can efficiently accelerate the water dissociation process, and thus V-CoP exhibits much better intrinsic catalytic activity. The result reveals that the V-CoP/CC electrode shows not only more active sites but also the higher intrinsic catalytic activity than CoP/CC. Remarkably, the present performance is at the top level among the previous reported CoP-based samples, as summarized in Figure 4F (Li et al., 2017; Ge et al., 2018).

**Figure 5:**
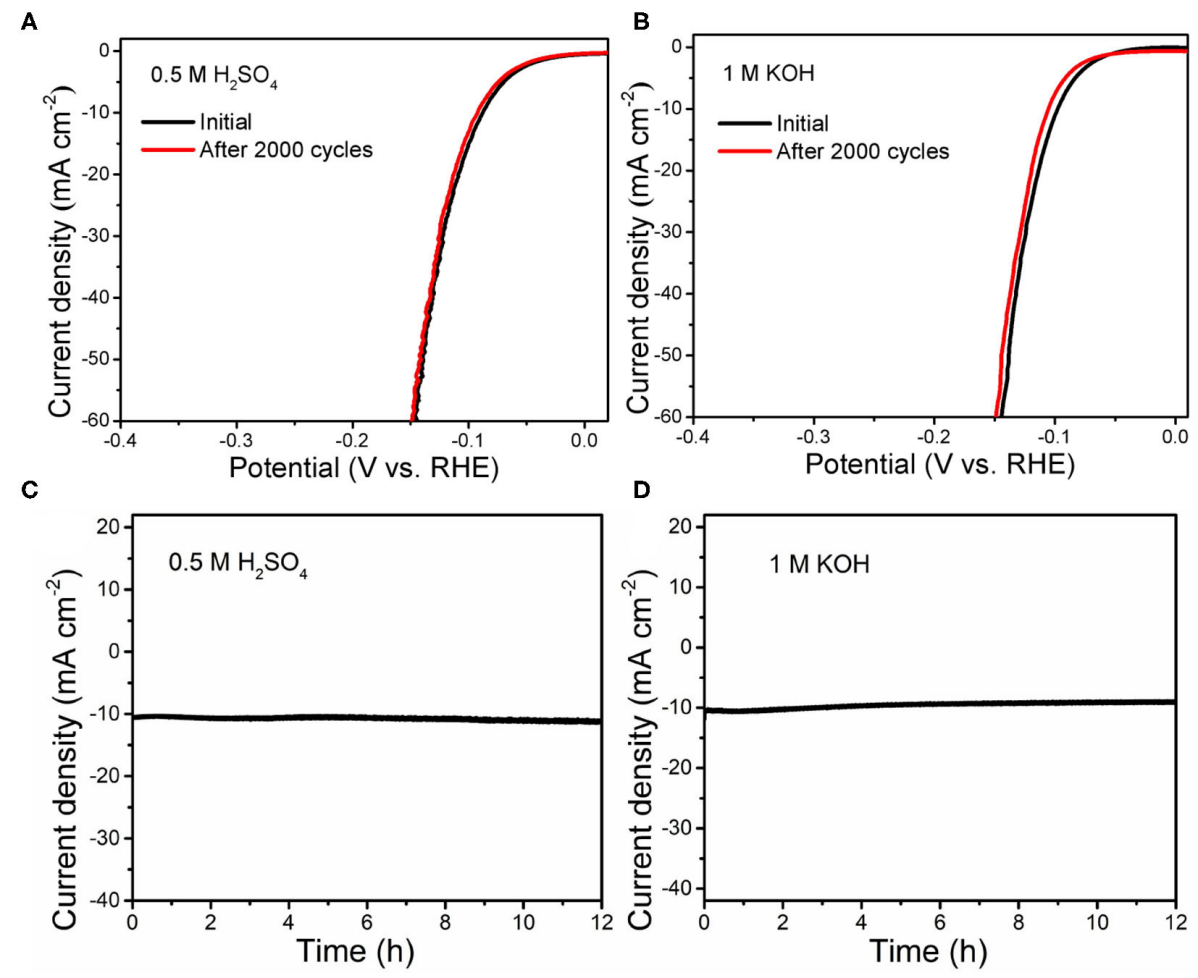
Polarization curves of V-CoP/CC initially and after 2,000 CV cycles **(A,B)** and time-dependent current density curves **(C,D)** in 0.5 M H_2_SO_4_ and 1.0 M KOH, respectively.

To evaluate the stability of V-CoP/CC in both acidic and basic solutions, long-term CV scanning is performed for 2,000 cycles. As displayed in Figure 5A, the polarization curves of the electrodes before and after the cycling test are almost overlapped under the acidic condition. As for the alkaline solution, only 8 mV negative shift is observed after the 2,000-cycling at the current density of 10 mA cm^−2^ (Figure 5B). In addition, the current density-time tests are carried out to explore the stability of the V-CoP/CC electrode. As shown in Figures 5C,D, the current density expresses a negligible decay during a 24-h durability test in 1 M KOH, while there is barely change in the current density during the measurement in 0.5M H_2_SO_4_. The above results show that the material has excellent stability in acidic media, and the performance has only a small attenuation under alkaline condition, which is mainly due to the generated corresponding hydroxides on the surface (Zhang et al., 2018; Lwu et al., 2019).

## Conclutions

In summary, a novel V-CoP nanosheet array grown on the CC skeleton has been successfully synthesized though a facile method. The V doping poses a positive effect on the electrocatalytic performance for HER under both acidic and alkaline conditions. The optimized electronic property by V doping is of great benefit in increasing the intrinsic catalytic activity of CoP. In addition, the nanoarray architecture is of great benefit for offering more accessible catalytic active sites. As a result, the V-CoP/CC electrode only requires small overpotentials of 88 and 98 mV to reach the current density of 10 mA cm^−2^ in acidic and basic media, respectively. The present hetero-atom doping strategy would offer a new window for rationally designing high active catalysts toward HER.

## Data Availability Statement

All datasets generated for this study are included in the article/Supplementary Material.

## Author Contributions

WH carried out experiments and wrote the paper. HS performed and analyzed experimental results. LR carried out experiments. DN designed experiments and contributed to writing the paper. All authors contributed to the article and approved the submitted version.

## Conflict of Interest

The authors declare that the research was conducted in the absence of any commercial or financial relationships that could be construed as a potential conflict of interest.

## References

[B1] Chen X. Wang Z. Wei Y. Zhang X. Zhang Q. Gu L.. (2019). High phase-purity 1T-MoS_2_ ultrathin nanosheets by a spatially confined template. Angew. Chem. Int. Ed. 58, 17621–17624. 10.1002/anie.201909879 31556194

[B2] Dong Z. Lin F. Yao Y. Jiao L. (2019). Crystalline Ni(OH)_2_/amorphous NiMoO_x_ mixed catalyst with Pt-like performance for hydrogen production. Adv. Energy Mater. 9:1902703 10.1002/aenm.201902703

[B3] Du H. Xia L. Zhu S. Qu F. Qu F. (2018). Al-doped Ni_2_P nanosheet array: a superior and durable electrocatalyst for alkaline hydrogen evolution. Chem. Commun. 54, 2894–2897. 10.1039/C7CC09445K 29497711

[B4] Fang G. Zhou J. Liang C. Pan A. Zhang C. Tang Y. (2016). MOFs nanosheets derived porous metal oxide-coated three-dimensional substrates for lithium-ion battery applications. Nano Energy 26, 57–65. 10.1016/j.nanoen.2016.05.009

[B5] Gao S. Yang M. Li S. Xia J. Mei J. Xie S. (2019). A phosphorus-doped carbon sphere supported CoP nanocatalyst for electrochemical hydrogen evolution. Sustain. Energy Fuels 3, 823–830. 10.1039/C8SE00498F

[B6] Ge Y. Chen J. Chu H. Dong P. Craig S. R. Ajayan P. M. (2018). Urchin-like CoP with controlled manganese doping toward efficient hydrogen evolution reaction in both acid and alkaline solution. ACS Sustain. Chem. Eng. 6, 15162–15169. 10.1021/acssuschemeng.8b03638

[B7] Guan C. Liu X. Elshahawy A. M. Zhang H. Wu H. Pennycook S. J.. (2017). Metal–organic framework derived hollow CoS_2_ nanotube arrays: an efficient bifunctional electrocatalyst for overall water splitting. Nanosc. Horiz. 2, 342–348. 10.1039/C7NH00079K 32260664

[B8] Guan C. Xiao W. Wu H. Liu X. Zang W. Zhang H. (2018). Hollow Mo-doped CoP nanoarrays for efficient overall water splitting. Nano Energy 48, 73–80. 10.1016/j.nanoen.2018.03.034

[B9] Guo L. Bai X. Xue H. Sun J. Song T. Zhang S.. (2020). MOF-derived hierarchical 3D Bi-doped CoP nanoflower eletrocatalyst for hydrogen evolution reaction in both acidic and alkaline media. Chem. Commun. 56, 7702–7705. 10.1039/C9CC09684A 32662801

[B10] Hua W. Sun H.-H. Xu F. Wang J.-G. (2020). A review and perspective on molybdenum-based electrocatalysts for hydrogen evolution reaction. Rare Met. 39, 335–351. 10.1007/s12598-020-01384-7

[B11] Huang X. Xu X. Li C. Wu D. Cheng D. Cao D. (2019). Vertical CoP nanoarray wrapped by N,P-Doped carbon for hydrogen evolution reaction in both acidic and alkaline conditions. Adv. Energy Mater. 9:1803970 10.1002/aenm.201803970

[B12] Li H. Zhao X. Liu H. Chen S. Yang X. Lv C.. (2018). Sub-1.5 nm ultrathin CoP nanosheet aerogel: efficient electrocatalyst for hydrogen evolution reaction at all pH values. Small 14:1802824. 10.1002/smll.201802824 30350551

[B13] Li J. Zheng G. (2017). One-dimensional earth-abundant nanomaterials for water-splitting electrocatalysts. Adv. Sci. 4:1600380. 10.1002/advs.201600380 28331791 PMC5357991

[B14] Li Y. Liu J. Chen C. Zhang X. Chen J. (2017). Preparation of NiCoP hollow quasi-polyhedra and their electrocatalytic properties for hydrogen evolution in alkaline solution. ACS Appl. Mater. Interfaces 9, 5982–5991. 10.1021/acsami.6b14127 28121122

[B15] Li Y. Niu S. Rakov D. Wang Y. Cabán-Acevedo M. Zheng S.. (2018). Metal organic framework-derived CoPS/N-doped carbon for efficient electrocatalytic hydrogen evolution. Nanoscale 10, 7291–7297. 10.1039/C8NR01811A 29632920

[B16] Liang H. Gandi A. N. Anjum D. H. Wang X. Schwingenschlogl U. Alshareef H. N. (2016). Plasma-assisted synthesis of NiCoP for efficient overall water splitting. Nano Lett. 16, 7718–7725. 10.1021/acs.nanolett.6b03803 27960455

[B17] Liu B. Zhao Y. F. Peng H. Q. Zhang Z. Y. Sit C. K. Yuen M. F.. (2017). Nickel–cobalt diselenide 3D mesoporous nanosheet networks supported on Ni foam: an all-pH highly efficient integrated electrocatalyst for hydrogen evolution. Adv. Mater. 29:1606521. 10.1002/adma.201606521 28262994

[B18] Lu X. F. Yu L. Zhang J. Lou X. W. D. (2019). Ultrafine dual-phased carbide nanocrystals confined in porous nitrogen-doped carbon dodecahedrons for efficient hydrogen evolution reaction. Adv. Mater. 31:1900699. 10.1002/adma.201900699 31168857

[B19] Luo B. Huang T. Zhu Y. Wang D. (2017). Glucose-derived carbon sphere supported CoP as efficient and stable electrocatalysts for hydrogen evolution reaction. J. Energy Chem. 26, 1147–1152. 10.1016/j.jechem.2017.08.013

[B20] Luo J. Im J. H. Mayer M. T. Schreier M. Nazeeruddin M. K. Park N. G.. (2014). Water photolysis at 12.3% efficiency via perovskite photovoltaics and earth-abundant catalysts. Science 345, 1593–1596. 10.1126/science.1258307 25258076

[B21] Lwu Z. Huang L. Liu H. Wang H. (2019). Element-specific restructuring of anion- and cation-substituted cobalt phosphide nanoparticles under electrochemical water-splitting conditions. ACS Catal. 9, 2956–2961. 10.1021/acscatal.8b03835

[B22] Pan Y. Liu Y. Lin Y. Liu C. (2016). Metal doping effect of the M-Co_2_P/nitrogen-doped carbon nanotubes (M = Fe, Ni, Cu) hydrogen evolution hybrid catalysts. ACS Appl. Mater. Interfaces 8, 13890–13901. 10.1021/acsami.6b02023 27197546

[B23] Seh Z. W. Kibsgaard J. Dickens C. F. Chorkendorff I. Nørskov J. K. Jaramillo T. F. (2017). Combining theory and experiment in electrocatalysis: insights into materials design. Science 355:eaad4998. 10.1126/science.aad4998 28082532

[B24] Shi Y. Zhang B. (2016). Recent advances in transition metal phosphide nanomaterials: synthesis and applications in hydrogen evolution reaction. Chem. Soc. Rev. 45, 1529–1541. 10.1039/C5CS00434A 26806563

[B25] Sun H. Liu H. Hou Z. Zhou R. Liu X. Wang J.-G. (2020). Edge-terminated MoS_2_ nanosheets with an expanded interlayer spacing on graphene to boost supercapacitive performance. Chem. Eng. J. 387:124204 10.1016/j.cej.2020.124204

[B26] Wang H. Min S. Wang Q. Li D. Casillas G. Ma C.. (2017). Nitrogen-doped nanoporous carbon membranes with Co/CoP janus-type nanocrystals as hydrogen evolution electrode in both acidic and alkaline environments. ACS Nano 11, 4358–4364. 10.1021/acsnano.7b01946 28362485

[B27] Wang J. G. Hua W. Li M. Liu H. Shao M. Wei B. (2018). Structurally engineered hyperbranched NiCoP arrays with superior electrocatalytic activities toward highly efficient overall water splitting. ACS Appl. Mater. Interfaces 10, 41237–41245. 10.1021/acsami.8b11576 30398830

[B28] Wang X. Chen Y. Yu B. Wang Z. Wang H. Sun B.. (2019). Hierarchically porous W-doped CoP nanoflake arrays as highly efficient and stable electrocatalyst for pH-universal hydrogen evolution. Small 15:1902613. 10.1002/smll.201902613 31361084

[B29] Wang Y. Kong B. Zhao D. Wang H. Selomulya C. (2017). Strategies for developing transition metal phosphides as heterogeneous electrocatalysts for water splitting. Nano Today 15, 26–55. 10.1016/j.nantod.2017.06.006

[B30] Wang Y. Sun Y. Yan F. Zhu C. Gao P. Zhang X. (2018). Self-supported NiMo-based nanowire arrays as bifunctional electrocatalysts for full water splitting. J. Mater. Chem. A 6, 8479–8487. 10.1039/C8TA00517F

[B31] Yao N. Li P. Zhou Z. Zhao Y. Cheng G. Chen S. (2019). Synergistically tuning water and hydrogen binding abilities over Co_4_N by Cr doping for exceptional alkaline hydrogen evolution electrocatalysis. Adv. Energy Mater. 9:1902449 10.1002/aenm.201902449

[B32] Zhang G. Wang B. Bi J. Fang D. Yang S. (2019). Constructing ultrathin CoP nanomeshes by Er-doping for highly efficient bifunctional electrocatalysts for overall water splitting. J. Mater. Chem. A 7, 5769–5778. 10.1039/C9TA00530G

[B33] Zhang J. Wang T. Liu P. Liao Z. Liu S. Zhuang X.. (2017). Efficient hydrogen production on MoNi_4_ electrocatalysts with fast water dissociation kinetics. Nat. Commun. 8:15437. 10.1038/ncomms15437 28513620 PMC5442356

[B34] Zhang Y. Gao L. Hensen E. J. M. Hofmann J. P. (2018). Evaluating the stability of Co_2_P electrocatalysts in the hydrogen evolution reaction for both acidic and alkaline electrolytes. ACS Energy Lett. 3, 1360–1365. 10.1021/acsenergylett.8b00514 29911183 PMC5996345

[B35] Zou X. Zhang Y. (2015). Noble metal-free hydrogen evolution catalysts for water splitting. Chem. Soc. Rev. 44, 5148–5180. 10.1039/C4CS00448E 25886650

