# Supplementary material for: V-Doped CoP Nanosheet Arrays as Highly Efficient Electrocatalysts for Hydrogen Evolution Reaction in Both Acidic and Alkaline Solutions
Source: Front Chem. 2020 Oct 23;8:608133. doi: 10.3389/fchem.2020.608133 (PMC7645198; doi:10.3389/fchem.2020.608133)
Supplement: Supplementary file 1 [file Data_Sheet_1.doc]

**V-Doped CoP Nanosheet Arrays as Highly Efficient Electrocatalysts for Hydrogen Evolution Reaction in Both Acidic and Alkaline Solutions**

**Wei Hua ^1^, Huanhuan Sun ^1^, Lingbo Ren ^1^, Ding Nan ^2,*^**

^1^ State Key Laboratory of Solidification Processing, Center for Nano Energy Materials, School of Materials Science and Engineering, Northwestern Polytechnical University, Shaanxi Joint Lab of Graphene (Northwestern Polytechnical University), Xi’an, China, ^2^ School of Materials Science and Engineering, Inner Mongolia University of Technology, Hohhot, China

*** Correspondence:**Corresponding Author: nan1980732@163.com (D. Nan)


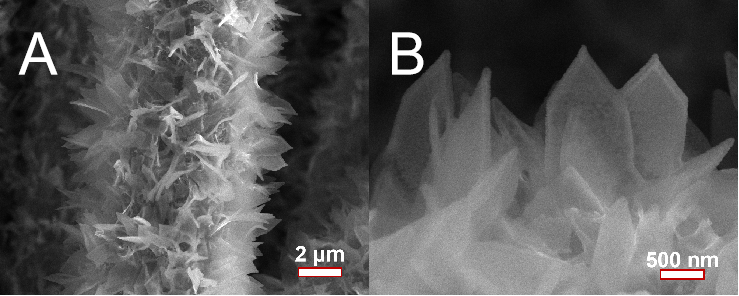


**FIGURE S1** **|** SEM images of CoP on the CC.

**FIGURE S2** **|** The XRD spectra of CoP/CC and V-CoP/CC

**FIGURE S3** **|** CV curves of (A) V-CoP and (B) CoP electrodes at difference scan rates in 0.5 M H_2_SO_4_.

**FIGURE S4** **|** CV curves of (A) V-CoP and (B) CoP electrodes at difference scan rates in 1 M KOH.
